# IL-33 reflects dynamics of disease activity in patients with autoimmune hemolytic anemia by regulating autoantibody production

**DOI:** 10.1186/s12967-015-0745-0

**Published:** 2015-12-16

**Authors:** Xiangmao Bu, Tenglong Zhang, Chunhong Wang, Tao Ren, Zhenke Wen

**Affiliations:** Department of Clinical Laboratory, Qingdao Women & Children Hospital, Qingdao, 266034 Shandong China; Department of Oncology, Qingdao Municipal Hospital, Qingdao, 266071 Shandong China; Department of Respiratory Medicine, East Hospital, Tongji University School of Medicine, 150 Jimo Road, Shanghai, 200120 China; Institute of Immunobiology, Shanghai Medical College of Fudan University, Shanghai, 200032 China; Division of Immunology and Rheumatology, Stanford University School of Medicine, 269 Campus Drive West, Stanford, CA 94305 USA

**Keywords:** Autoimmune hemolytic anemia, IL-33, Red blood cell, Anti-RBC autoantibody

## Abstract

**Background:**

Autoimmune hemolytic anemia (AIHA), a life-threatening anemia with rapid onset, is caused by autoantibody directed to self red blood cells (RBCs). Currently, mechanisms underlying AIHA pathogenesis are largely undefined. Here we explored the correlation of IL-33 with AIHA disease activity and evaluated IL-33 based therapeutics in AIHA treatment.

**Methods:**

Thirty patients diagnosed with AIHA of warm-type autoantibodies without treatment were enrolled and followed up for 6 months. Levels of cytokines including IL-33, IL-4, IL-6 and IL-13 was determined with ELISA. AIHA disease activity was presented by levels of reticulocyte count, hemoglobin and lactate dehydrogenase. Serum RBC-bound IgG autoantibody was detected using anti-IgG antibody with flow cytometry. To evaluate the effect of IL-33 blockade on AIHA development, groups of B6 mice were immunized with rat RBCs plus recombinant IL-33 protein or IL-33 neutralizing antibody respectively and detected for levels of anti-RBC antibody, frequency of reticulocytes and destruction of transfused syngeneic mouse RBCs.

**Results:**

Serum level of IL-33 was higher in AIHA patients compared with healthy individuals. Of interest, serum IL-33 was positively correlated with AIHA disease activity and sensitive to their changes in AIHA patients under clinical management. Mechanistically, IL-33 could promote the production of anti-RBC autoantibody. Serum IL-33 was closely associated with serum anti-RBC autoantibody and sensitive to their changes in AIHA patients. Accordingly, blockade of IL-33 interfered with AIHA incidence and ameliorated disease activity. Vice vasa, enforced IL-33 promoted AIHA incidence and disease activity.

**Conclusions:**

IL-33 was a potential biomarker for monitoring disease activity and therapeutic response in AIHA patients. Targeting IL-33 was a promising strategy for controlling autoantibody production in AIHA patients.

**Electronic supplementary material:**

The online version of this article (doi:10.1186/s12967-015-0745-0) contains supplementary material, which is available to authorized users.

## Background

Autoimmune hemolytic anemia (AIHA), a life-threatening anemia with rapid onset, is defined by enhanced destruction of red blood cells (RBCs) because of the production and accumulation of anti-RBC autoantibodies [1, 2]. AIHA can be classified by optimal temperature at which the anti-RBC autoantibodies bind to a patient’s RBCs, and warm AIHA accounts for about 80 % of all AIHA cases [3]. In patients with warm AIHA, the anti-RBC autoantibodies are usually IgG type and bind optimally to RBCs at 37 °C, causing RBC destruction by tissue macrophages [1, 4–6]. Currently, therapeutic options for patients with AIHA are still limited due to the obscure pathogenesis.

Interleukin 33 (IL-33), a novel identified member of IL-1 family, can be produced in various tissues and cells, and orchestrate complex innate and adaptive immune responses [7–9]. Through binding to the orphan receptor ST2, IL-33 can induce gene expression of Th2-associated cytokines, promote Th2 type immune response, and thus play important roles in autoimmune diseases [9–11]. In rheumatoid arthritis, a higher level of IL-33 was detected in synovial fluid, serum, and was correlated with disease activity compared to moderate or low activity group or healthy controls [12, 13]. Blockade of IL-33/ST2 interaction resulted in dramatically attenuated disease severity of rheumatoid arthritis [14]. In systemic lupus erythematosus, elevated serum IL-33 was reported in clinical patients and correlated with their disease activity [15–17]. Disrupting IL-33 pathway resulted in reduced serum anti-dsDNA levels, alleviated proteinuria, and attenuated lupus nephritis in lupus-prone mice [18]. These findings demonstrated a possible role of IL-33 in production of IgG autoantibodies and development of autoimmune diseases. However, whether IL-33 functions in AIHA pathogenesis still remains unclear.

In this study, we investigated the potential role of IL-33 in AIHA pathogenesis in patients with warm AIHA. Our findings revealed an elevated level of serum IL-33 in patients with AIHA. Of interest, serum IL-33 was correlated with disease activity and sensitive to their changes in AIHA patients. IL-33 could facilitate production of IgG anti-RBC autoantibody from peripheral blood mononuclear cells in AIHA patients. Blockade of IL-33 restrained AIHA incidence and disease activity, while enforced IL-33 promoted the development of AIHA. These findings assigned IL-33 an important function involved in AIHA pathogenesis and provided clues for exploring new AIHA therapeutics.

## Methods

### Patients

Thirty patients diagnosed with AIHA of warm-type autoantibodies without treatment were enrolled and followed up for 6 months in this study. AIHA diagnosis was made with clinical and laboratory hemolytic signs plus positive direct anti-globulin test (DAT) as previously described [1]. Eighteen age- and sex-matched healthy controls were studied. Patients were considered as having active disease when an anemia and at least one of the following were present: reticulocyte count >2 %, haptoglobin concentration <500 mg/L and lactate dehydrogenase (LDH) activity >480 U/L. All blood samples and clinical parameters were collected after an informed consent. This study was approved by the Ethics Committee of Tongji University and performed in accordance with the ethical standards laid down in the 1964 Declaration of Helsinki and its later amendments.

### Cell culture and reagents

Peripheral blood mononuclear cells (PBMCs) were isolated from heparinized peripheral blood using Ficoll-Paque density gradient (GE Healthcare). PBMCs were washed with phosphate-buffered saline (PBS) and cultured in RPMI 1640 medium with 10 % FCS and 1 % glutamine/penicillin/streptomycin (Life Technologies). Recombinant human IL-33 protein was purchased from R&D Systems. Anti-human IgM antibody was purchased from eBioscience. Recombinant human CD40 Ligand (CD40L) was purchased from Life Technologies. For in vitro induction of anti-RBC IgG antibody, PBMCs (1 × 10^6^/ml) were cultured with anti-IgM (10 μg/ml) and CD40L (10 ng/ml) in the presence or absence of IL-33 (0–20 ng/ml). Six days later, the supernatants were collected and assayed for IgG antibodies with Human IgG total ELISA kit (eBioscience).

### Cytokines

Serum level of IL-33 was determined with Human IL-33 Quantikine ELISA Kit (R&D Systems). IL-4, IL-6 and IL-13 levels in culture supernatants were also detected with Human IL-4, IL-6 and IL-13 Quantikine ELISA Kits (R&D Systems) respectively according to the manual’s instructions.

### Anti-RBC IgG antibody

Serum RBC-bound IgG autoantibody was detected with flow cytometry as described previously [1, 19]. Briefly, RBCs were freshly isolated, washed three times with warm PBS and then incubated with FITC anti-IgG antibody (Biolegend) at 37 °C. The cells were immediately analyzed for percentage of RBC-bound antibody on a FACSCalibur flow cytometer (BD Biosciences). Collected data were analyzed with FlowJo software (TreeStar).

### Induction of murine AIHA

Murine experiments were approved by the Ethics Committee of Tongji University. Female B6 mice between 8 and 10 weeks old were purchased from Shanghai Laboratory Animal Center and housed in specific pathogen free conditions. As previously described [1], induction of AIHA murine model was achieved by immunizing B6 mice with 2 × 10^8^ rat RBCs on a weekly basis for 10 weeks. For transfusion study, mouse RBCs obtained from naive female B6 mice were labeled with PKH-26 (Sigma) and injected into control mice or those that had developed AIHA through tail-vein. Destruction of these syngeneic RBCs was represented by the clearance of fluorescent RBCs measured by flow cytometry. To show the clearance kinetics, injected RBCs at 1 min after injection were taken as 100 %, and the remaining RBCs were calculated at different time points as the average for each group of mice.

For regulation of IL-33, B6 mice were injected with IL-33 neutralizing antibody or recombinant IL-33 respectively with isotype controls (R&D Systems). Briefly, 1 day before rat RBCs immunization, mice were intraperitoneally injected with 100 μg neutralizing antibodies per mouse or 2 μg IL-33 per mouse respectively. Injections were repeated every 3 days during mice immunization.

### Statistical analyses

Quantitative data were expressed as the mean ± SD. Unpaired t test and Pearson correlation were used for statistical analyses with PRISM 6.0 (GraphPad Software Inc.). A value of P < 0.05 was considered statistically significant.

## Results

### Serum level of IL-33 was elevated and correlated with disease activity in AIHA patients

**Fig. 1:**
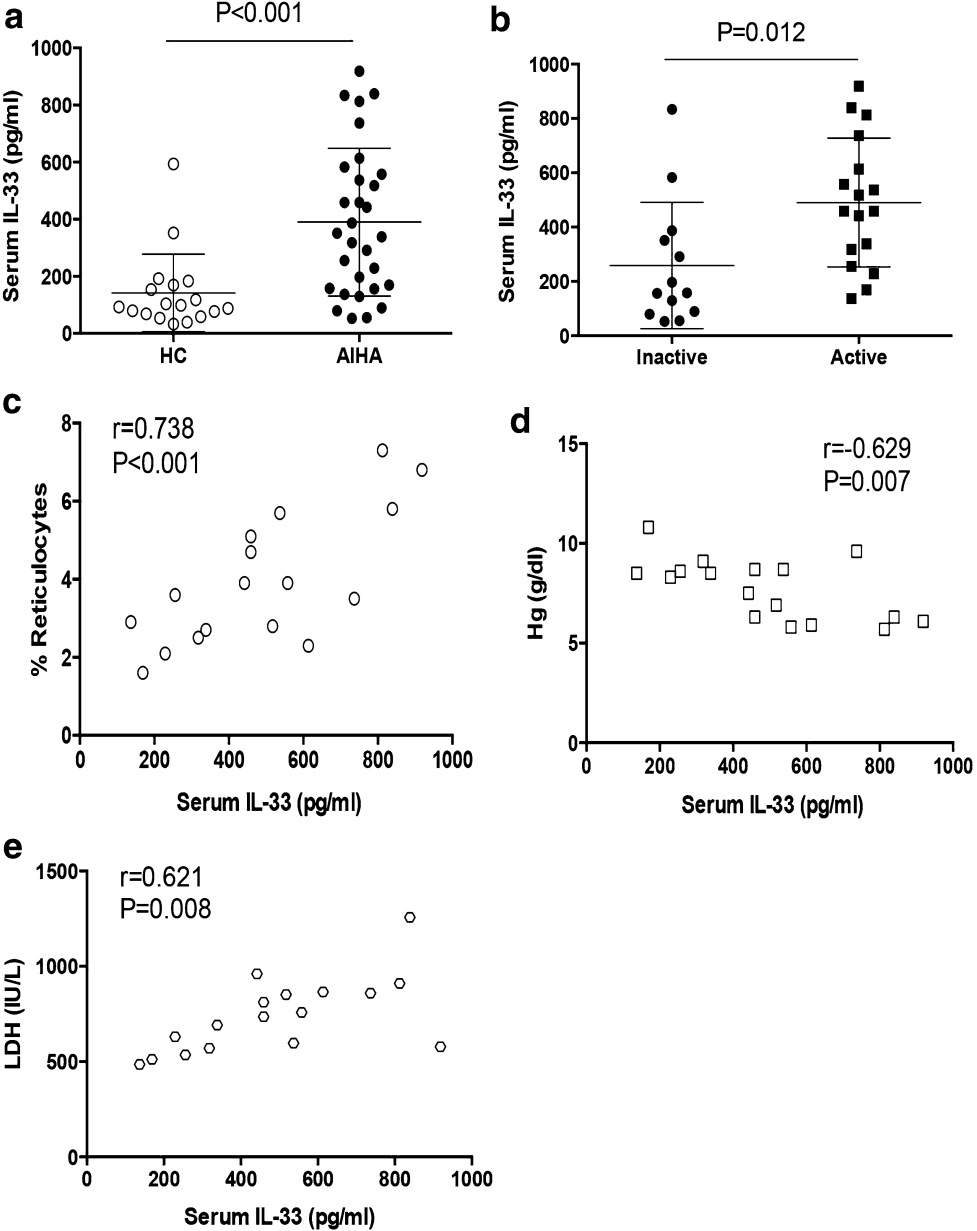
Elevated serum IL-33 in AIHA patients was correlated with AIHA disease activity. **a** Serum IL-33 was determined in AIHA patients (n = 30) and healthy controls (n = 18); **b** serum IL-33 was detected in patients with active AIHA (n = 17) or with inactive AIHA (n = 13); **c**–**e** the correlation between serum IL-33 and levels of reticulocyte count (**c**), hemoglobin (**d**) and lactate dehydrogenase (LDH, **e**) was analyzed in active AIHA patients (n = 17). One dot represented the result from one patient

We performed ELISA to determine serum levels of IL-33 in patients with AIHA and healthy controls, and results showed a significant higher level of serum IL-33 in AIHA patients (Fig. 1a). Further analysis of serum IL-33 in patients with active AIHA or with AIHA remission revealed a relative higher serum IL-33 in patients with active AIHA (Fig. 1b). These data indicated an involvement of IL-33 in AIHA pathogenesis. Thus, we detected the correlation between serum IL-33 and disease activity of active AIHA patients that is presented by the levels of reticulocyte count, hemoglobin and lactate dehydrogenase (LDH). We found that serum level of IL-33 was closely correlated with the level of reticulocyte frequency, negatively correlated with the level of hemoglobin and positively correlated with the level of LDH activity in AIHA patients (Fig. 1c–e). These results demonstrated that serum IL-33 was closely correlated with disease activity of AIHA.

### Serum IL-33 was sensitive to changes of AIHA disease activity

**Fig. 2:**
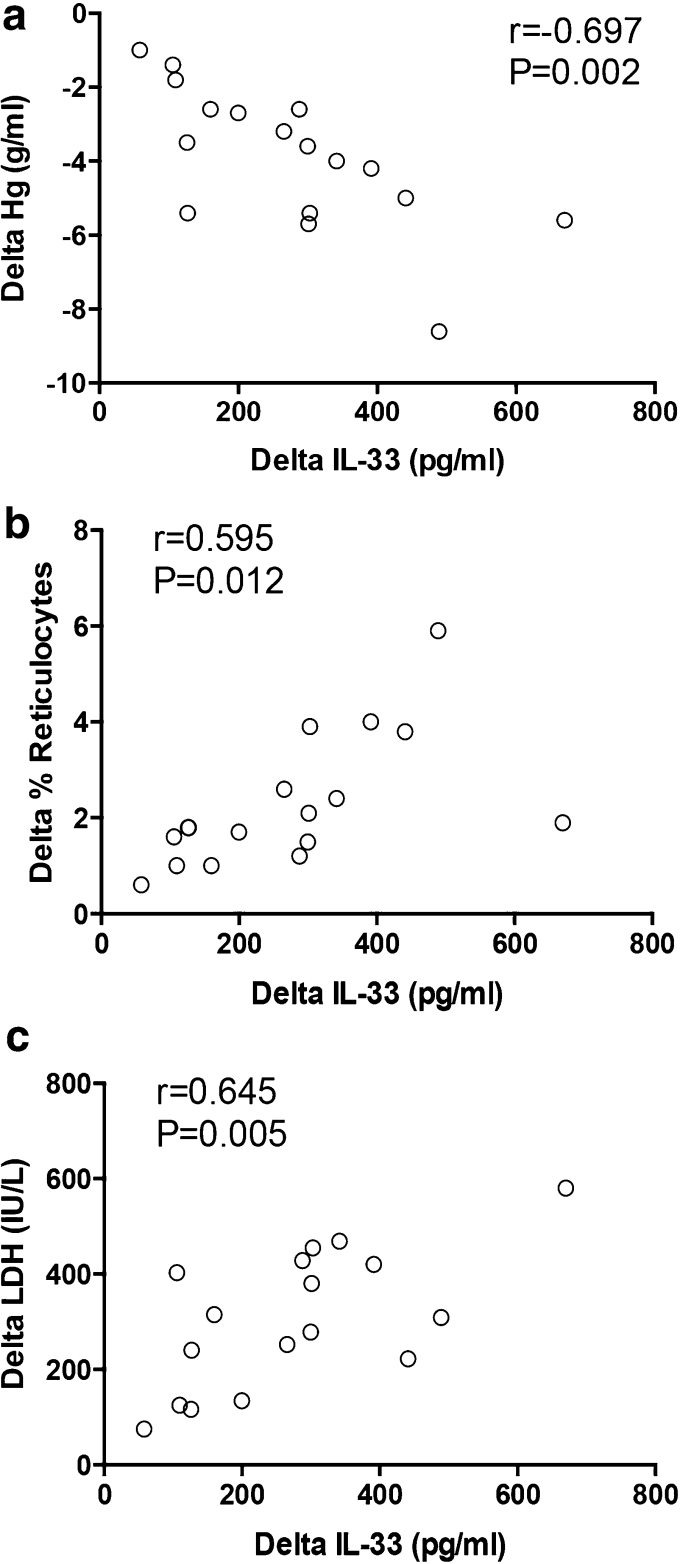
IL-33 was sensitive to AIHA disease activity. Patients with active AIHA (n = 17) were followed up and analyzed for the changes of serum IL-33, reticulocyte count, hemoglobin and LDH. *Delta* represented the different values between post-follow and pre-follow. The correlation between changes of serum IL-33 and changes of hemoglobin (**a**), changes of reticulocyte count (**b**), and changes of LDH (**c**) was detected. One *dot* represented the result from one patient

To further explore the correlation of serum IL-33 with AIHA disease activity, patients with active AIHA were followed up for 6 months and analyzed for their changes of serum IL-33 and disease activity. Results showed that changes of serum IL-33 were associated with the changes of hemoglobin, positively correlated with changes of reticulocyte frequency and LDH in AIHA patients (Fig. 2a–c). These findings suggested that serum IL-33 was sensitive to changes of AIHA disease activity.

### Serum IL-33 was associated with anti-RBC autoantibody production

**Fig. 3:**
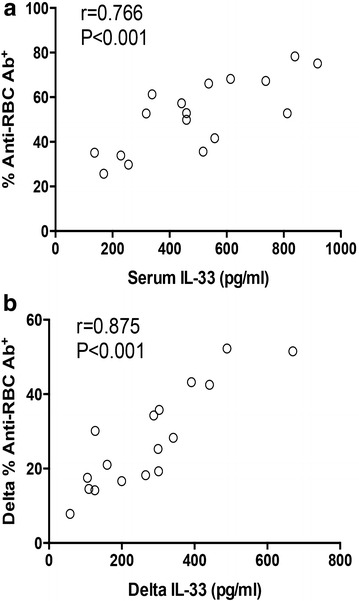
IL-33 was associated with anti-RBC antibody production. **a** The correlation of serum IL-33 with anti-RBC antibody level was determined in AIHA patients (n = 17); **b** the correlation between changes of serum IL-33 and changes of anti-RBC antibody production was analyzed in AIHA patients (n = 17). One *dot* represented the result from one patient

To investigate the possible mechanisms underlying the close relationship between serum IL-33 and disease activity of AIHA patients, we analyzed the correlation of serum IL-33 with anti-RBC autoantibody, which plays a central role in AIHA pathogenesis. We found that serum IL-33 was positively correlated with the anti-RBC autoantibody level in AIHA patients (Fig. 3a). Further, changes of serum IL-33 was closely associated with changes of anti-RBC autoantibody (Fig. 3b). These results could partly explain the close correlation of serum IL-33 with AIHA disease activity and indicated an involvement of IL-33 in autoantibody production in AIHA patients.

### IL-33 contributed to anti-RBC autoantibody production

**Fig. 4:**
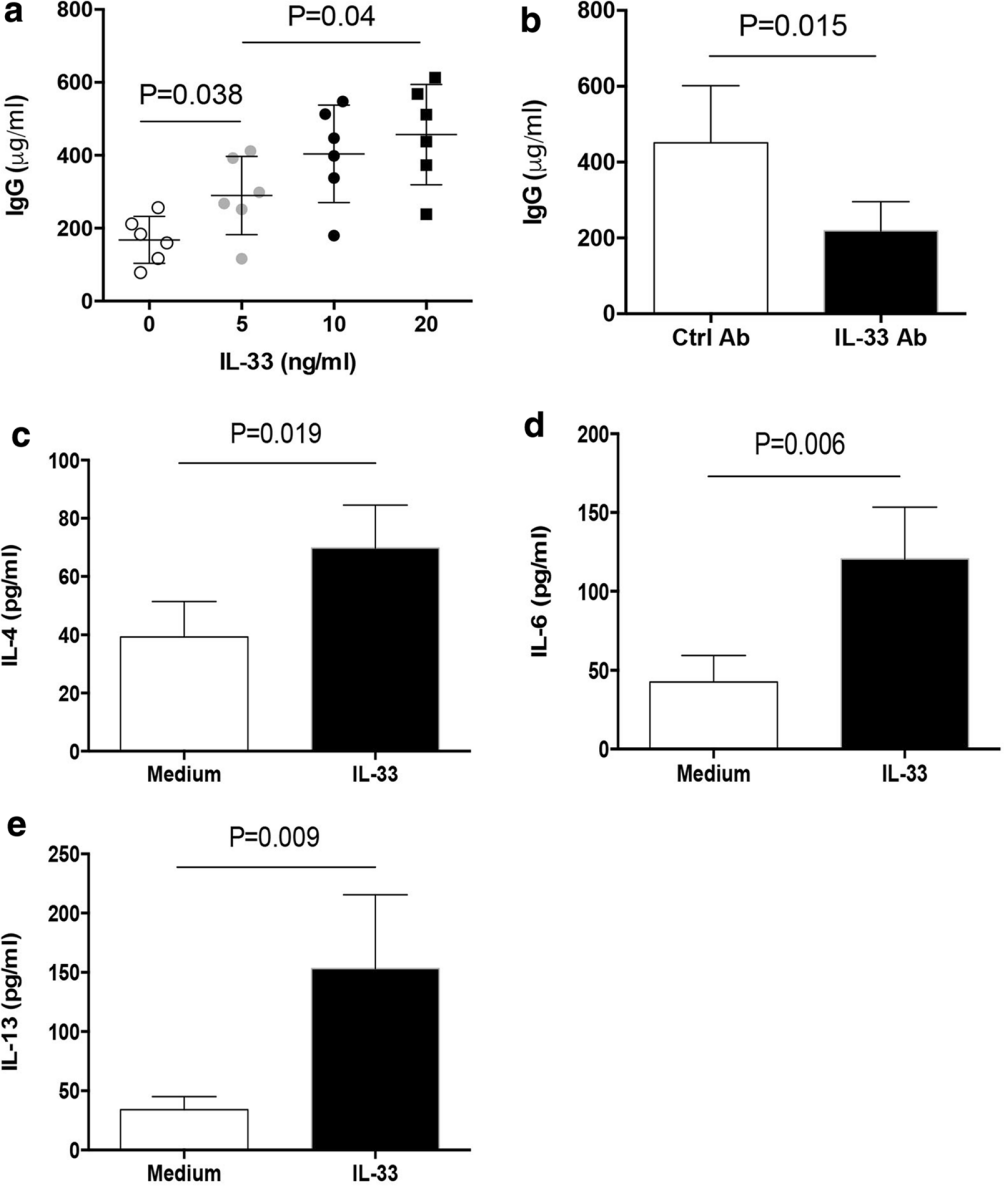
IL-33 promoted anti-RBC antibody production. **a** PBMCs from AIHA patients (n = 6) were stimulated with anti-IgM, CD40L in the presence of indicated dose of IL-33 for 6d, and then assayed for IgG antibody production. One dot represented the result from one patient; **b** PBMCs from AIHA patients (n = 5) were stimulated with anti-IgM, CD40L plus IL-33 (20 ng/ml) in the presence of IL-33 neutralizing antibody or control antibody (2 μg/ml) for 6d, and detected for IgG antibody; **c**–**e** PBMCs from AIHA patients (n = 4) were stimulated with IL-33 (20 ng/ml) for 72 h, followed by detection for production of IL-4, IL-6 and IL-13 in supernatants

To further elucidate why serum IL-33 was correlated with anti-RBC autoantibody level, we detected the effect of IL-33 on IgG anti-RBC antibody production in AIHA patients. When PBMCs isolated from patients with active AIHA were stimulated with anti-IgM plus CD40L in the presence of an increasing dose of recombinant human IL-33 protein, we found that IL-33 could promote the production of IgG anti-RBC antibody in a dose dependent manner (Fig. 4a). Vice vasa, blockade of IL-33 efficiently reduced the enhanced production of IgG anti-RBC antibody (Fig. 4b). Given the important roles of IL-4, IL-6 and IL-13 in antibody production, we further detect the production of these cytokines in response to IL-33 stimulation. We found that stimulation of PBMCs isolated from active AIHA patients with IL-33 protein resulted in significant higher production of IL-4, IL-6 and IL-13 (Fig. 4c–e). These findings demonstrated that IL-33 could increase Th2 cytokines release and contribute to autoantibody production in patients with AIHA.

### Blockade of IL-33 interfered with incidence and disease severity of AIHA

**Fig. 5:**
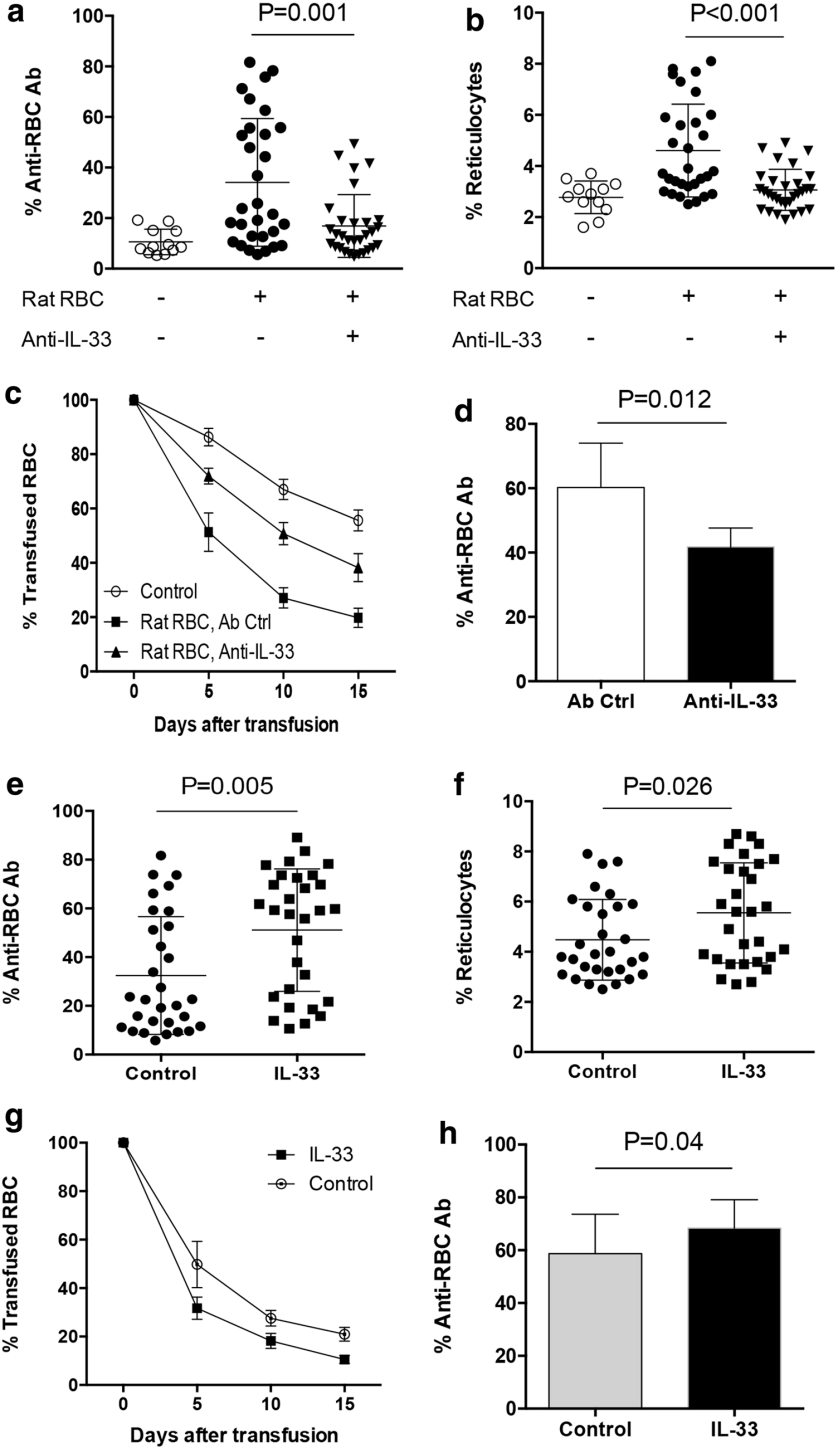
IL-33 neutralization restrained AIHA development. **a**–**c** Female B6 mice (n = 30) were injected with neutralizing antibody to IL-33 or the isotype control, and immunized with rat RBCs for 10 weeks. Then the level of anti-RBC IgG antibody, frequency of circulating reticulocytes and the clearance kinetics of transfused syngeneic mouse RBCs were determined. Each *dot* represented the results from one mouse. Each *bar* represented the collective data from control mice (n = 7), IL-33 treated AIHA mice (n = 5) and Ab Ctrl group (n = 11). **d** The level of anti-RBC IgG antibody in AIHA mice co-injected of IL-33 neutralizing antibody (n = 5) or isotype control (n = 13). Each *dot* represented the results from one AIHA mouse. **e**–**g** Female B6 mice (n = 30) were immunized with rat RBCs plus IL-33 for 10 weeks, and detected for the level of anti-RBC IgG antibody, frequency of circulating reticulocytes and the clearance kinetics of transfused syngeneic mouse RBCs. Each *dot* represented the results from one mouse. Each *bar* represented the collective data from five mice. **h** Serum level of IgG anti-RBC antibody in AIHA mice with (n = 19) or without (n = 12) recombinant IL-33 injection. Each *bar* represented the collective data (mean ± SD) from each group

To directly evaluate the potential role of IL-33 in AIHA pathogenesis, murine AIHA was induced by immunizing B6 mice with rat RBCs with or without IL-33 neutralizing antibody. Within 30 mice immunized with rat RBCs, 13 mice co-injected with isotype control IgG exerted high levels of anti-RBC antibody and reticulocytes (Additional file 1: Fig. S1, 5A, B). Consistent with this observation, these mice showed increased destruction of transfused syngeneic mouse RBCs (Fig. 5c). When B6 mice were co-injected with IL-33 neutralizing antibody, only 5 out of 30 mice developed AIHA as evidenced by the circulating levels of anti-RBC antibody and reticulocytes (Fig. 5a, b). Of note, the level of anti-RBC antibody was significantly decreased by co-injection with IL-33 neutralizing antibody (Fig. 5d). Thus, the destruction of transferred syngeneic mouse RBCs was inhibited in IL-33 neutralizing antibody co-immunized mice (Fig. 5c). To further confirm the effect of IL-33 on AIHA pathogenesis, groups of B6 mice were immunized with rat RBCs plus recombinant IL-33 protein. Results showed that enforced IL-33 significantly enhanced the incidence of AIHA and elevated the generation of IgG anti-RBC antibody (Fig. 5e–h). These results regarded IL-33 as an important regulator in AIHA pathogenesis and suggested that blockade of IL-33 was a promising strategy to control AIHA disease.

## Discussion

AIHA is an autoimmune disorder caused by production of autoantibody that directed against self red blood cells [20, 21]. Therefore, intervention of anti-RBC autoantibody production is essential for AIHA treatment. Currently, therapeutic options for AIHA are usually corticosteroids, splenectomy, intravenous immunoglobulins, plasma-exchange and other immunosuppressive drugs, which are non-specific and have considerable side effects [2, 22–24]. Rituximab, a monoclonal antibody against the protein CD20 and can destroy immune system B cells, has been tested in treatment of AIHA [3, 25–27]. However, autoantibody production in AIHA, especially IgG autoantibody that accounts for warm AIHA development, requires involvement of T helper cells for B cell responses. Thus, modulation of T cell responses could be an optimal strategy for AIHA treatment.

IL-33 is a newly described cytokine of IL-1 superfamily and is reported to be involved in T cell mediated immune responses [15]. Herein, we reported that serum IL-33 was positively associated with and sensitive to anti-RBC autoantibody levels in AIHA patients. In contrast, serum levels of sST2 in AIHA patients were generally comparable with that in healthy individuals (Additional file 1: Fig. S2), conferring an active function of serum IL-33 in AIHA patients. Further, IL-33 could promote the release of Th2 cytokines and the production of IgG anti-RBC autoantibody. Thus, accumulation of IL-33 in peripheral blood could facilitate the anti-RBC autoantibody production, which provided a new mechanism through which the autoantibody production was exacerbated in AIHA patients. Therefore, it is reasonable to speculate that IL-33 might be a promising target for controlling autoantibody production and thus restrains AIHA development. In support, we demonstrated that IL-33 could promote the development of AIHA, and more importantly, blockade of IL-33 effectively reduced the incidence of AIHA and ameliorated AIHA disease severity. These results extended previous studies that implicated IL-33 based therapeutics in treatment of a range of diseases including autoimmune disease [9, 15].

Herein, we revealed that serum IL-33 was significantly elevated in patients with AIHA and closely correlated with the disease activity. Our results were consistent with previous studies that showed elevated serum IL-33 in autoimmune diseases [28–30]. The increased levels of IL-33 in AIHA patients might result from destructive RBCs and macrophages because RBCs could store and release IL-33, and macrophages served as important scavengers [31, 32]. Our findings suggested that serum level of IL-33 might serve as a potential biomarker for AIHA disease activity. Besides, serum IL-33 was sensitive to changes of disease activity and anti-RBC autoantibody production in AIHA patients, indicating that serum IL-33 was also a promising indicator for monitoring the efficiency of clinical practice. To our knowledge, this is the first study that described the elevated serum IL-33 and its close correlation with disease activity in AIHA patients. Considering the facts that around 5 % of AIHA remains DAT-negative and treatment of AIHA is currently not evidence-based [33], new biomarkers that have diagnostic values or that can be used to assess treatment response could be helpful in clinical practice.

In this study, we observed an interesting effect of IL-33 on anti-RBC antibody production, which might due to increased secretion of Th2 cytokines. In fact, we found diminished levels of Th2 cytokines in AIHA mice co-injected with anti-IL-33 neutralizing antibody, and elevated levels Th2 cytokines in AIHA mice co-injected with IL-33 (Additional file 1: Fig. S3A, B). These Th2 cytokines are considered important for supporting B cell responses. We observed elevated frequency of B cells within PBMCs after stimulation with IL-33 in vitro (Additional file 1: Fig. S4A), and defective development of germinal center B cells in AIHA mice co-injected with anti-IL-33 neutralizing antibody (Additional file 1: Fig. S4B). Further, disrupt IL-6 signaling with neutralizing antibody significantly abrogated the effect of IL-33 on IgG anti-RBC antibody production (Additional file 1: Fig. S5). These findings suggested important roles of IL-33-mediated Th2 cytokines in AIHA pathogenesis. It is well acknowledged that ST2 is highly expressed on group 2 innate lymphoid cells and T helper 2 cells [34]. Therefore, we proposed that production of anti-RBC antibody in AIHA patients was, at least in part, dependent on ST2^+^ T cells, which released increased levels of Th2 cytokines in response to IL-33 and thus support B cell responses. The destructive RBCs and engaged macrophages would promote the accumulation of IL-33 and feed-forward the generation of anti-RBC antibody in AIHA patients.

## Conclusions

Here we reported an elevated serum IL-33 and its contribution to anti-RBC autoantibody production in patients with AIHA. IL-33 was a potential biomarker for monitoring disease activity and treatment response in AIHA patients. Targeting IL-33 was a promising therapeutic strategy for AIHA patients in clinical practice.

## Additional file

10.1186/s12967-015-0745-0 Gating strategy for anti-RBC antibody analysis, serum levels of sST2 in AIHA patients, Th2 cytokines in AIHA mice co-injected with IL-33 protein or IL-33 neutralizing antibody, B cell response to IL-33 stimulation or IL-33 blockade, and effect of IL-6 on IL-33-mediated autoantibody production were presented.

## Abbreviations

AIHA: autoimmune hemolytic anemia
RBC: red blood cell
IL-33: interleukin 33
PBMC: peripheral blood mononuclear cells
LDH: lactate dehydrogenase
