# Supplementary material for: IL-33 reflects dynamics of disease activity in patients with autoimmune hemolytic anemia by regulating autoantibody production
Source: J Transl Med. 2015 Dec 16;13:381. doi: 10.1186/s12967-015-0745-0 (PMC4681137; doi:10.1186/s12967-015-0745-0)
Supplement: Supplementary file 1 — 10.1186/s12967-015-0745-0 Gating strategy for anti-RBC antibody analysis, serum levels of sST2 in AIHA patients, Th2 cytokines in AIHA mice co-injected with IL-33 protein or IL-33 neutralizing antibody, B cell response to IL-33 stimulation or IL-33 blockade, and effect of IL-6 on IL-33-mediated autoantibody production were presented. [file 12967_2015_745_MOESM1_ESM.pdf]

Fig. S1

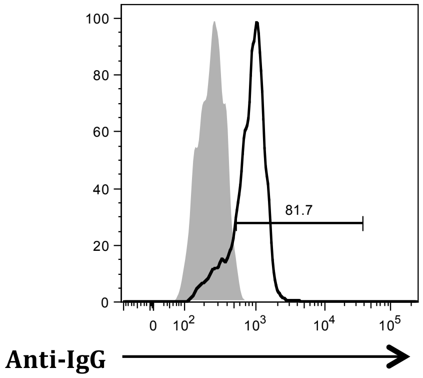

RBCs were freshly isolated from AIHA mice, washed three times with warm PBS and stained with FITC anti-IgG antibody (black) or control antibody (grey). Frequency of RBC-bound antibody was analyzed with flow cytometry. One representative image was shown.

Fig. S2

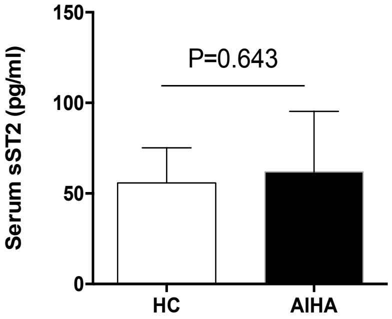

Serum level of soluble ST2 was detected in healthy controls (n=8) and AIHA patients (n=13).

Fig. S3

A

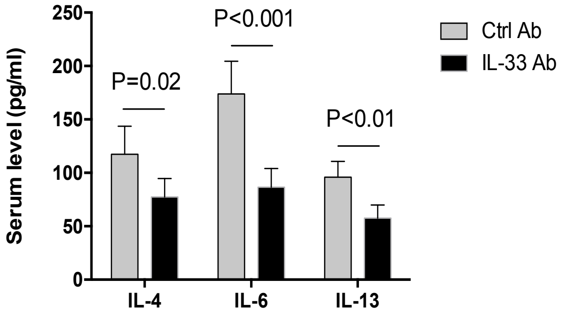

B

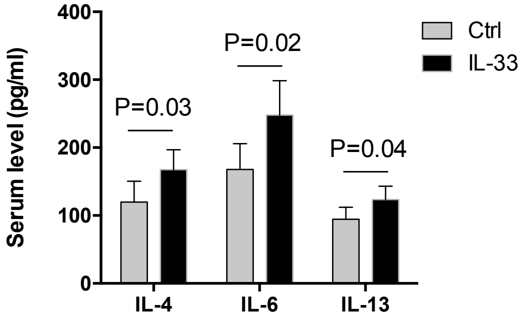

(A) Serum level of the indicated Th2 cytokines in AIHA mice (n=5) that were injected with neutralizing antibody to IL-33 or the control antibody and immunized with rat RBCs for 10 weeks.

(B) Serum level of the indicated Th2 cytokines in AIHA mice (n=5) that were injected with or without recombinant IL-33 protein and immunized with rat RBCs for 10 weeks.

Fig. S4

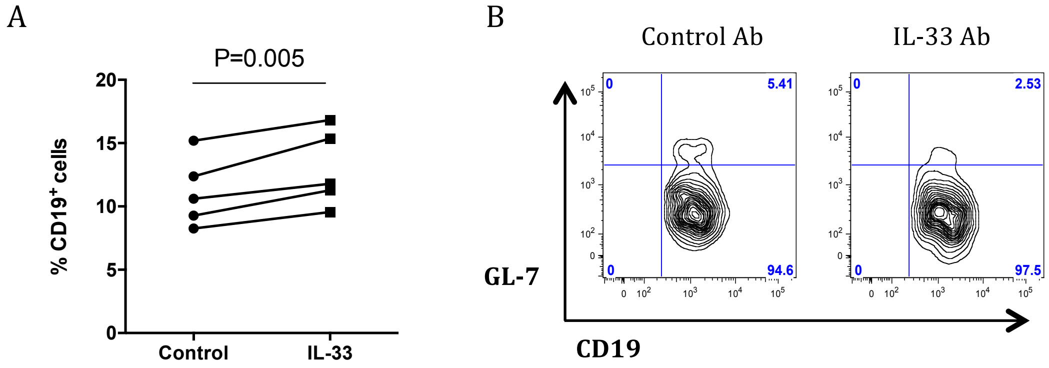

(A) PBMCs of AIHA patients (n=5) were stimulated with anti-IgM and CD40L with or without IL-33 for 6d, and analyzed for frequency of B cells.

(B) Female B6 mice were injected with neutralizing antibody to IL-33 or the control antibody, immunized with rat RBCs for 4 weeks, and detected for frequency of $CD19^+GL\text{-}7^+$ germinal center B cells in gated B cells within their splenocytes.

Fig. S5

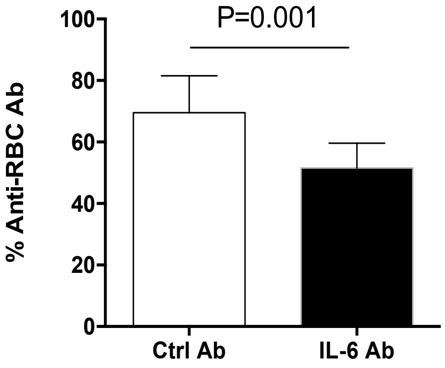

Serum levels of IgG anti-RBC antibody in AIHA mice (n=10) that were injected with IL-33 protein, immunized with rat RBCs in the presence or absence of IL-6 neutralizing antibody for 10 weeks.
